# Network Analysis of Gut Microbiome and Metabolome to Discover Microbiota-Linked Biomarkers in Patients Affected by Non-Small Cell Lung Cancer

**DOI:** 10.3390/ijms21228730

**Published:** 2020-11-19

**Authors:** Pamela Vernocchi, Tommaso Gili, Federica Conte, Federica Del Chierico, Giorgia Conta, Alfredo Miccheli, Andrea Botticelli, Paola Paci, Guido Caldarelli, Marianna Nuti, Paolo Marchetti, Lorenza Putignani

**Affiliations:** 1 Area of Genetics and Rare Diseases, Unit of Human Microbiome, Bambino Gesù Children’s Hospital, IRCCS, 00146 Rome, Italy; pamela.vernocchi@opbg.net (P.V.); federica.delchierico@opbg.net (F.D.C.); 2 IMT School for Advanced Studies Lucca, Networks Unit, 55100 Lucca, Italy; tommaso.gili@imtlucca.it; 3 Institute for Systems Analysis and Computer Science “Antonio Ruberti”, National Research Council, 00185 Rome, Italy; federica.conte@iasi.cnr.it; 4 Department of Chemistry, NMR-Based Metabolomics Laboratory Sapienza, University of Rome, 00185 Rome, Italy; giorgia.conta@uniroma1.it; 5 Department of Environmental Biology and NMR-Based Metabolomics Laboratory, Sapienza University of Rome, 00185 Rome, Italy; alfredo.miccheli@uniroma1.it; 6 Department of Clinical and Molecular Medicine, Sapienza University of Rome, 00185 Rome, Italy; andrea.botticelli@uniroma1.it (A.B.); paolo.marchetti@uniroma1.it (P.M.); 7 AOU Policlinico Umberto I, 00161 Rome, Italy; 8 Department of Computer, Control and Management Engineering, Sapienza University of Rome, 00185 Rome, Italy; paci@diag.uniroma1.it; 9 Department of Molecular Sciences and Nanosystems, Ca’ Foscari, University of Venice, 30172 Venice, Italy; guido.caldarelli@unive.it; 10 European Centre for Living Technologies, 30172 Venice, Italy; 11 Institute of Complex Systems (CNR), Department of Physics, University of Rome “Sapienza”, 00185 Rome, Italy; 12 Department of Experimental Medicine, University Sapienza of Rome, 00185 Rome, Italy; marianna.nuti@uniroma1.it; 13 AOU Sant’ Andrea Hospital, 00189 Rome, Italy; 14 Department of Diagnostic and Laboratory Medicine, Unit of Parasitology and Area of Genetics and Rare Diseases, Unit of Human Microbiome, Bambino Gesù Children’s Hospital, IRCCS, 00165 Rome, Italy

**Keywords:** non-small cell lung cancer (NSCLC), anti-PD1 immune checkpoint inhibitor, gut microbiome, operational taxonomic unit (OTU), metabolite, network analysis, weighted gene co-expression network analysis (WGCNA), betweenness centrality, clustering coefficient, communities

## Abstract

Several studies in recent times have linked gut microbiome (GM) diversity to the pathogenesis of cancer and its role in disease progression through immune response, inflammation and metabolism modulation. This study focused on the use of network analysis and weighted gene co-expression network analysis (WGCNA) to identify the biological interaction between the gut ecosystem and its metabolites that could impact the immunotherapy response in non-small cell lung cancer (NSCLC) patients undergoing second-line treatment with anti-PD1. Metabolomic data were merged with operational taxonomic units (OTUs) from 16S RNA-targeted metagenomics and classified by chemometric models. The traits considered for the analyses were: (i) condition: disease or control (CTRLs), and (ii) treatment: responder (R) or non-responder (NR). Network analysis indicated that indole and its derivatives, aldehydes and alcohols could play a signaling role in GM functionality. WGCNA generated, instead, strong correlations between short-chain fatty acids (SCFAs) and a healthy GM. Furthermore, commensal bacteria such as *Akkermansia muciniphila*, Rikenellaceae, *Bacteroides*, Peptostreptococcaceae, Mogibacteriaceae and Clostridiaceae were found to be more abundant in CTRLs than in NSCLC patients. Our preliminary study demonstrates that the discovery of microbiota-linked biomarkers could provide an indication on the road towards personalized management of NSCLC patients.

## 1. Introduction

The gut microbiota (GM) plays a fundamental role in the functional framework of the host immune system and any changes in diet, overuse of antibiotics, xenobiotics and gastrointestinal (GI) tract infections can lead to substantial shifts in the ecology and composition of the individual microbiome over extensive periods [1]. Conditions such as immune and inflammatory disorders or induced immunosuppression lead to these shifts, a phenomenon called “dysbiosis” [2]. This imbalance alters cellular pathways or signals in some bacteria, causing inflammation, one of the salient characteristics of carcinogenesis. Thus, GM is considered integral in the development of different cancers such as colorectal, hepatocellular and breast cancer [3].

Chen and Mellman [4] described the significance of the GM in anti-cancer response via the concept of a cancer immunity cycle. Most cancers can evade detection of tumor antigens, resulting in a T_reg_ response rather than an effector T cell response. Proteins like Cytotoxic T-Lymphocyte Antigen 4 (CTLA-4) and Programmed cell death protein 1/Programmed death-ligand 1 (PD-1/PD-L1) have been identified as negative immune regulators or immune checkpoints (ICIs). They are represented as the main targets for immuno-therapeutic interventions. Even though immunotherapies with ICIs, namely anti-CTLA4 and anti-PD1, looked highly promising, they have shown only 20–30% success in treating cancer patients [5]. In order to obtain a successful stand in cancer immunotherapy, the therapeutic efforts must be focused on the initiation or re-initiation of the cancer immunity cycle, resulting in an unfettered immune response.

Several studies demonstrated that the GM can have a long-term effect on immune response, which could determine the efficacy of ICIs [6,7,8,9]. The microbes present in the GI tract can modulate the immune response via interaction with pattern recognition receptors (PRR) and subsequent signaling cascades [9]. Landmark studies in animal models revealed that gut microbes can directly affect the response to immunotherapy [10,11]. In humans, studies on cancer patients undergoing immunotherapy revealed a higher microbial diversity correlated with improved survival rates [12,13].

Lung cancer is the second most common cancer in men and women worldwide, with the highest incidence of morbidity and mortality [14]. Non-small cell lung cancer (NSCLC) accounts for more than 80% of all lung cancers and it is one of the most challenging to treat with a poor response to ICIs in most patients. Interestingly, likewise to the role of the GM in other cancers, recent findings also indicate a relationship between the GM and the lungs [15]. Moreover, there is growing evidence demonstrating the association of the GM and its metabolome in ICI treatment response in NSCLC [16,17,18,19]. Indeed, not only the microbial cells but also their metabolites play a role in the stimulation of the immune response [5]. Their interplays elicit and boost the immune response, aiding the host immune system in its fight against cancer.

In our previous studies [16,17], we analyzed the GM in responder and non-responder NSCLC patients undergoing anti-PD1 treatment. Our findings reported a higher incidence of Rikenellaceae, *Prevotella*, *Streptococcus*, *Lactobacillus*, *Bacteroides plebeius*, *Oscillospira* and Enterobacteriaceae in the stool of NSCLC patients compared to healthy controls. Besides, *Ruminococcus bromii*, *Dialister* and *Sutterella* species were observed to be abundant in the non-responder compared to responder patients [16]. Furthermore, an examination of the gut metabolome profile in these patients shed light on certain metabolites that could play a significant role in immune response [17]. The presence of short-chain fatty acids (SCFAs) (i.e., propionic, butyric, acetic, valeric acids), lysine and nicotinic acid indicated a better response to therapy, whereas metabolites such as tridecane and 2-pentanone were suggestive of non-responsiveness [17]. Hence, the composition of the GM and metabolome is a promising predictive biomarker for immunotherapy response.

In this study, we further investigated the GM ecological and metabolomic profile in NSCLC patients undergoing treatment with the anti-PD1 Nivolumab. Given the complexity in structure, function and compositional variability, the human GM can be modeled and represented as networks in order to deduce the dynamic nature of host–microbe interactions [20]. Network science is a powerful tool that uses mathematical models to analyze and comprehend complex systems [21]. In microbiome studies, network theory can be applied to understand different microbial communities, their metabolites and their inter-relationships, as well as their effects on the host’s health. Indeed, such studies give insights into probable molecular mechanisms occurring in different diseases, including cancer.

Here, network-based approaches using *network analysis* and *weighted gene co-expression network analysis* (WGCNA) were carried out to identify the central players that participate in the host GM signaling pathways and to find potential correlations with NSCLC condition and anti-PD1 treatment response.

## 2. Results and Discussion

### 2.1. Patient Characterization

For the study, 11 NSCLC patients and 8 normal healthy CTRLs were recruited. Of the 11 NSCLC patients, 4 patients did not respond to immunotherapy and were considered as non-responder. The other seven patients were categorized as responder (R) (test results/i-RECIST/radiology results) [17]. In particular, we defined non-responder (NR) patients experiencing disease progression within three months from the beginning of nivolumab [17].

### 2.2. Weighted Gene Co-Expression Network Analysis (WGCNA) Analysis

We used the well-established WGCNA framework [22,23] as the data exploration method (i.e., to investigate data related to the two traits of interest, NSCLC/CTRLs and R/NR) and as the feature selection method (i.e., for screening the most important features).

**Figure 1:**
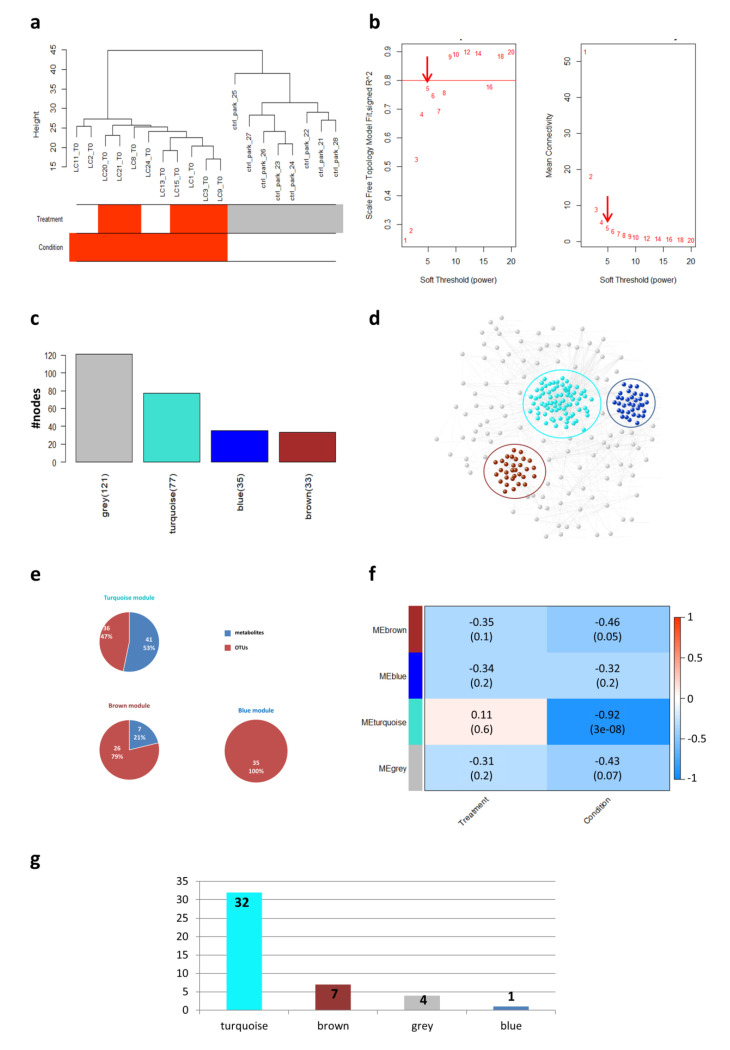
Weighted Gene Co-expression Network Analysis (WGCNA). (**a**) Clustering dendrogram of samples. Sample clustering was conducted to detect outliers. All samples were located in the clusters and passed the cut-off thresholds. The horizontal bars represent how the traits (i.e., condition and response to treatment) relate to the sample dendrogram: white (low value) represents controls (CTRLs) and non-responder (NR) for condition and treatment traits, respectively; red (high value) represents case and responder (R) for condition and treatment traits, respectively; gray means missing entry. (**b**) Calculation and selection of optimal soft-thresholding rule. Influence of different powers on the scale independence (left) and on the mean connectivity (right). The red arrow indicates the selected soft-thresholding power. (**c**) Barplot. The bars represent the size of each WGCNA-detected module and were colored according to the corresponding module labels. (**d**) Weighted correlation network. In the network, the WGCNA-detected modules were highlighted with a colored circle according to the corresponding module labels. Nodes in the network are colored according to the corresponding module labels. (**e**) Pie charts. Pie charts represent the percentages and numbers of operational taxonomic units (OTUs) and metabolites falling in each WGCNA-detected module. (**f**) Module–traits associations. In the heatmap, each row corresponds to a module eigengene (ME) and each column to a trait. Each cell contains the corresponding correlation and *p*-value. The table is color-coded by correlation according to the color legend. (**g**) Distribution of features (OTUs and metabolites) identified at the end of the data processing, in the WGCNA-detected modules.

We firstly applied WGCNA on the dataset obtained by merging metagenomics operational taxonomic units (OTUs) (Supplementary Table S1) and metabolomics (gas chromatography (GC–MS) and nuclear magnetic resonance (NMR) spectroscopy) matrices (Supplementary Tables S2 and S3), just considering patients’ R/NR response to therapy (Supplementary Text S1, Figures S1 and S2 and Table S4). Since these results were not statistically significant, only the NSCLC/CTRLs condition was subsequently considered. Therefore, the WGNCA analysis was performed just onOTUs and GC–MS data (Figure 1) because the NMR analysis did not include CTRLs samples.

Initially, a sample clustering was conducted to detect outliers, and all the samples in the cluster passed the cut-off thresholds (Figure 1a). The clustering algorithm identified two main clusters: one corresponding to patients and the other one corresponding to CTRLs. Interestingly, these results showed that the metagenomics and metabolomics data clearly distinguished patients from controls (Figure 1a).

The weighted network was built using the Pearson correlation matrix and the soft-thresholding power was set equal to 5 (Figure 1b). The final network consisted of four modules (labeled by color), ranging in size from 33 to 121 members (Figure 1c–e). The gray module represented the grouping of nodes with outlying profiles and was not considered henceforth. The values for module membership (MM) and biomarker significance (BS) and the corresponding *p*-values were calculated for each node in each module, as reported in Supplementary Table S5.

Finally, tests of association between traits and the module eigengenes (MEs) were performed for each module and the results were summarized in a heatmap (Figure 1f). The only statistically significant correlations were found with respect to the NSCLC/CTRLs variable. In particular, the turquoise module appeared to show the highest correlation with the condition.

The majority of the features in the turquoise module had a negative sign of the correlation value (Supplementary Table S5_turquoise, BS condition), suggesting that the OTUs and metabolites falling in this module were mainly expressed in controls rather than in the disease condition. The metabolites that showed a higher correlation with CTRLs were represented by: acids (pentanoic, butyric), aldehydes (benzaldehyde, benzenacetaldehyde, 3-methyl-butanal, 2-butenal), ketones (acetone, 2-heptanone, 2-octanone), terpenes (g-terpinene, 3-carene) and *p*-cresol (Supplementary Table S5_turquoise). On the other hand, the OTUs linked to CTRLs (*p* ≤ 0.05) were represented by: Rikenellaceae, *Bacteroides caccae* and *Dialister.* Moreover, the turquoise module indicated positive correlation values (*p* ≤ 0.05) for certain metabolites, mainly expressed in patients, including dodecane, 2,6-dimethyl-4 heptanone and methyl isobutyl ketone (Supplementary Table S5_turquoise).

In the brown module, microbes such as Peptostreptococcaceae, Mogibacteriaceae, Clostridiaceae and Coriobacteriaceae and the metabolite anethole showed a negative sign of the correlation value (Supplementary Table S5_brown, BS condition), implying an association with CTRLs. On the other hand, the bacterium *Granulicatella* was found to be significantly higher in responder patients (Supplementary Table S5_brown, BS treatment). This bacterium could therefore be used as a possible predictive biomarker for anti-PD1 treatment response.

The blue module was represented by 100% of OTUs, suggesting a highly interconnected set of microorganisms in the gut. Microbial species like Peptococcaceae and Prevotellaceae demonstrated a higher expression in the CTRLs group since they showed a negative sign of the correlation value (Supplementary Table S5_blue, BS condition).

In general, the WGCNA analysis revealed that changes in the GM and metabolome were mainly associated with the NSCLC/CTRLs condition. Furthermore, we carried out other network-based analyses focusing only on features whose expression changed with statistical significance between the NSCLC and CTRLs conditions (Supplementary Figure S3 and Table S6). Notably, 73% of these selected features fell in the turquoise module, thus representing the main features associated with the NSCLC/CTRLs status in the WGCNA network (Figure 1g).

The observations from the WGCNA analysis were in accordance with the literature, emphasizing the association of SCFAs, such as pentanoic and butyric acids, in healthy gut [24]. Moreover, they represent beneficial products derived from both proteolytic and saccharolytic fermentation [25] used not only as an energy source for the host’s colonocytes, but also for their anti-inflammatory properties [26,27]. SCFAs are involved in immune system regulation and they are generally known as having a role in tumor suppression [28].

In lung cancer studies, a different pool of metabolites, i.e., ketones, esters, aldehydes and alcohols, were considered in order to discriminate among groups of patients [17]. However, the specific biological matrices affected both the qualitative and quantitative profiles of the final set of possible biomarkers [29,30].

Indeed, amongst VOCs identified in stool samples, metabolites like terpenes (g-terpinene and 3-carene) showed a statistical significance even though they might not be directly correlated with the condition as they originate from dietary sources (fruits and vegetables) [31].

Methyl phenol, also called *p*-cresol, is produced via microbial degradation of tyrosine. It is metabolized at the liver level and excreted in urine as *p*-cresol sulphate [25]. *p*-Cresol was also suggested as a biomarker of protein intake [32].

Moreover, *Clostridium difficile*, among other gut bacteria, showed its ability to produce *p*-cresol as a bacteriostatic compound through fermentation of tyrosine [33].

Since all these features resulted higher in CTRLs, it was possible to hypothesize that they might be associated with age and diet habits as it has been shown that in older people, the GM composition could be affected by an altered absorption of nutrients, digestion modifications and weakened immune system [34].

In addition, diet changes may cause a decrease in GM diversity, for example, a decrease in anaerobic bacteria such as *Bifidobacterium* spp. and an increase in *Clostridium* have been observed [34].

Rikenellaceae, *Bacteroides,* Peptostreptococcaceae, Mogibacteriaceae and Clostridiaceae showed a higher correlation with CTRLs than NSCLC, suggesting dysbiosis in lung cancer patients, as detected by other authors [16].

The current study did not yield statistically significant information regarding the correlation of features (OTUs and metabolites) between patients who were anti-PD1 R and NR. However, the highlight of these aspects was the association of the OTU *Granulicatella* with Nivolumab treatment response [35]. An additional study on a larger cohort size will be required to confirm this. Continued research in this area is necessary to further examine the key participants in the GM of the NSCLC immunotherapy response network.

### 2.3. Network Analysis

After metagenomics and metabolomics profiling, 198 OTUs, 223 VOCs and 49 non-volatile metabolites were identified (Supplementary Tables S1 and S2). In order to select the relevant microbes and metabolites for the network analysis, data were further processed.

**Figure 2:**
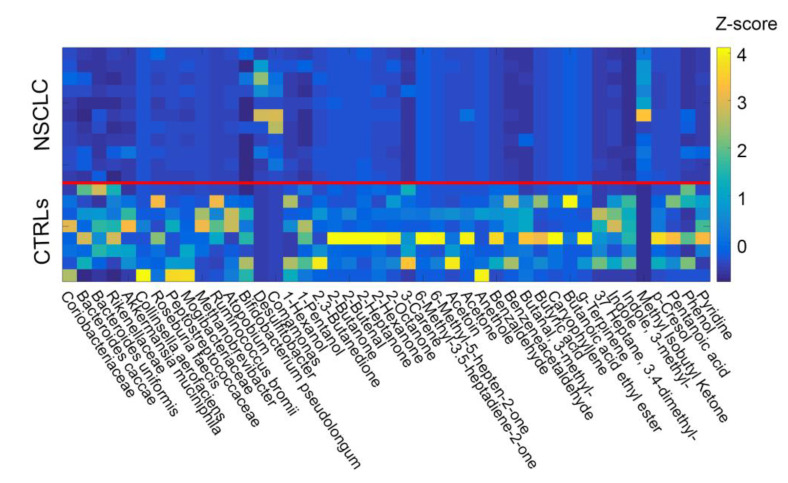
Z scores across subjects (NSCLC = 1–11, CTRLs = 12–19) calculated for the selected elements of the two groups (OTUs and metabolites). At the end of the filtration procedure, forty-four compounds were identified and included in the analysis. Data from patients and controls were merged and cross-correlated. The solid red line helps to separate the two groups (NSCLC and CTRLs).

**Figure 3:**
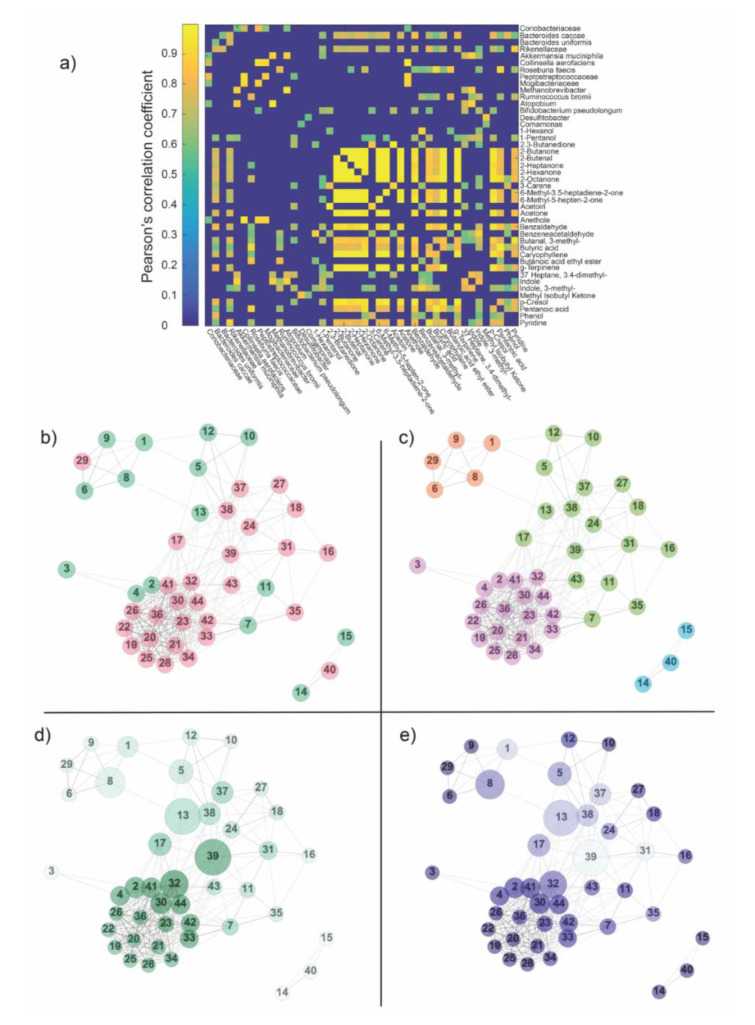
(**a**) The matrix obtained by cross-correlating OTUs and metabolites across subjects was thresholded according to statistical significance (*p* < 0.05) and False Discovery Rate (FDR)-corrected for multiple comparisons. Only positive correlations were found to be statistically significant and subsequently associated with the edges, giving place to the weighted adjacency matrix, i.e., the network. (**b**) The distribution of features within the network: green—OTUs, pink—metabolites. Numbers within the nodes identify features according to the order shown aside the weighted adjacency matrix. The network was divided into two main components: a big one and a triplet (nodes 14, 15, 40). (**c**) Communities founded by the Louvain algorithm in the network. Colors were used only for distinguishing the different communities, which, in turn, represents the affinity of species according to their covariance across subjects. (**d**) Degree and betweenness centrality calculated in the network. The size of each node is proportional to its betweenness, while the color code (from white to dark green) refers to the magnitude of the degree centrality (from low degree to high degree). (**e**) Clustering coefficient and betweenness centrality calculated in the network. The size of each node is proportional to its betweenness centrality, while the color code (from white to dark blue) refers to the magnitude of the clustering coefficient (from low clustering to high clustering coefficient). Legend Panel b–e. (1) Coriobacteriaceae; (2) *Bacteroides caccae*; (3) *Bacteroides uniformis*; (4) Rikenellaceae; (5) *Akkermansia muciniphila*; (6) *Collinsella aerofaciens*; (7) *Roseburia faecis*; (8) Peptostreptococcaceae; (9) Mogibacteriaceae; (10) *Methanobrevibacter*; (11) *Ruminococcus bromii*; (12) *Atopobium*; (13) *Bifidobacterium pseudolongum*; (14) *Desulfitobacter*; (15) *Comamonas*; (16) 1-Hexanol; (17) 1-Pentanol; (18) 2.3-Butanedione; (19) 2-Butanone; (20) 2-Butenal; (21) 2-Heptanone; (22) 2-Hexanone; (23) 2-Octanone; (24) 3-Carene; (25) 6-Methyl-3.5-heptadiene-2-one; (26) 6-Methyl-5-hepten-2-one; (27) Acetoin; (28) Acetone; (29) Anethole; (30) Benzaldehyde; (31) Benzeneacetaldehyde; (32) Butanal, 3-methyl-; (33) Butyric acid; (34) Caryophyllene; (35) Butanoic acid ethyl ester; (36) g-Terpinene; (37) Heptane, 3.4-dimethyl-; (38) Indole; (39) Indole, 3-methyl-; (40) Methyl Isobutyl Ketone; (41) *p*-cresol; (42) Pentanoic acid; (43) Phenol; (44) Pyridine.

At the end of the data pre-processing, forty-four compounds among bacteria and metabolite features were qualified to be included in the network analysis (Figure 2). The two datasets were merged together (Supplementary Table S7) and a correlation matrix was obtained cross-correlating all the features against one another. Figure 3a represents the final matrix after filtration by False Discovery Rate (FDR) correction and removal of disconnected nodes. Only positive correlations were found to be within the threshold value and subsequently associated with the edges, giving place to the weighted adjacency matrix, i.e., the network.

Once the network was obtained, some global and local topological properties were estimated. The global properties evaluated here included the distribution of features in the networks (Figure 3b, Supplementary Table S7) and the communities (Figure 3c, Supplementary Table S7). Meanwhile, the local properties of the networks that were analyzed included degree and betweenness centrality (Figure 3d, Supplementary Table S7) and clustering coefficient (Figure 3e, Supplementary Table S7).

From a global perspective, the network was divided into two main components: a giant one and a triplet (Figure 3b). The small three-nodes component was found to be composed of a metabolite, node 40 (methyl isobutyl ketone) and two OTUs, node 14 and 15 (*Desulfitobacter* and *Comamonas*). The community detection algorithm subdivided the network into four modules (Figure 3c): one represented by the three-nodes component and three coming from the giant components. By comparing Figure 3b,c, the participation ratio (number of OTUs/number of metabolites) in the four communities resulted to be: orange module = 0.25, light blue module = 0.5, violet module ~5 and green module ~2. The modules found by the algorithm essentially represent the affinity of features according to their covariance across subjects.

Figure 3d,e show the local properties of the network: degree centrality, betweenness centrality and clustering coefficient. Nodes 39 and 13, which represent the metabolite 3-methyl indole and OTU *Bifidobacterium pseudolongum***,** showed a high degree and betweenness centrality (Figure 3d), suggesting that they could be necessary for network functionality. On the contrary, 3-methyl indole was characterized by a low value of the clustering coefficient, suggesting that probably it did not allow for discriminating between NSCLC and CTRLs. Another key observation was node 32, namely 3-methyl butanal, which expressed a high degree and clustering co-efficient in the network (Figure 3e). This could suggest this node as a hub of connection among the violet module and the other nodes in the network, thus serving as an another important signaling molecule to maintain balance in the gut ecosystem.

Other nodes of interest (Figure 3d,e) were found to be the OTUs Peptostreptococcaceae (node 8) and *Akkermansia muciniphila* (node 5) and metabolites 1-pentanol (node 17), 3.4-dimethyl heptane (node 37) and indole (node 38). All the cited features resulted higher in CTRLs compared to NSCLC (Supplementary Tables S1 and S2).

Consistent with earlier studies, our results emphasize that bacteria such as *Bifidobacterium* and *Akkermansia muciniphila,* were some of the main players in the GM network functionality, usually indicated as the signature of a healthy intestinal ecosystem [17,36]. Indeed, *A. muciniphila* is a mucin-degrading bacterium that is known to help maintain gut mucosa and host health. *Akkermansia muciniphila* could improve liver activity, alleviate oxidative stress, normalize gut microbes and suppress inflammation. Thus, it might also be considered as one of the new probiotics [37].

Tryptophan catabolites like indole and indole derivatives have shown to exert anti-inflammatory and anti-oxidative effects in the gut by activating aryl hydrocarbon receptor signaling in astrocytes suppressing central nervous system inflammation, contributing to intestinal and systemic homeostasis [38]. Numerous bacteria including species of *Bacteroides, Clostridium, Lactobacillus, Peptostreptococcus, Bifidobacterium* and *Ruminococcus* have been demonstrated to be involved in the degradation of tryptophan into several catabolites [39]. In addition, indole 3-methyl, also known as skatole, is a well-known gut metabolite generated by the decarboxylation of indoleacetic acid by *Bacteroides* spp. and *Clostridium* spp.

Finally, butanal is a common aldehyde in the gut formed by bacterial breakdown of leucine, and high levels of this metabolite have been associated with inflammation and cancer [40].

However, the precise mechanisms of action and cellular signaling pathways involved in NSCLC are yet to be delineated.

## 3. Materials and Methods

### 3.1. Selection of Patients and Controls

The cohort of 11 NSCLC patients was recruited at the Department of Clinical and Molecular Medicine, Sant’ Andrea Hospital, Sapienza University of Rome, for 1 year from 2016 to 2017. The study group included 8 male and 3 female patients, between 44 to 82 years of age, with a median age of 68 years.

The inclusion criteria for patient selection included (i) age >18 years; (ii) histology diagnosis of NSCLC; (iii) Eastern Cooperative Oncology Group (ECOG) performance status ≤2; (iv) anti-PD-1 nivolumab as second-line treatment; and (v) satisfactory pulmonary, cardiac, liver, renal and bone marrow functions. Meanwhile, the medical conditions considered for exclusion criteria were (i) autoimmune diseases; (ii) symptomatic interstitial lung disease and other comorbidities; (iii) systemic immunosuppression; and (iv) precedent treatment with an immune-stimulatory antitumor factor such as checkpoint-targeted molecules.

Nivolumab (anti-PD1) was prescribed to patients at a dosage of 3 mg/kg every 2 weeks until disease progression or toxicity was observed. Using the Response Evaluation Criteria in Solid Tumors (i-RECIST) criteria, radiological response was assessed. Toxicity evaluation was conducted on day 1 of every dosage cycle until the end of cure. Toxicity, if any, was reported in accordance with National Cancer Institute Common Terminology Criteria for Adverse Events (version 4.0). For our study, progression-free survival (PFS) represented the time from patient registration on clinical trial until the first recorded tumor progression or death from any cause, while the overall survival (OS) was the time from patient registration to death from whatever cause. Patients that showed tumor progression within 3 months from the starting of nivolumab treatment were considered as non-responders (NR), whereas patients presenting PFS longer than 12 months were defined as responders (R).

The study also included a cohort of 8 CTRLs, who were age-matched with the patients. They were screened by a survey conducted by the Human Microbiome Unit at Bambino Gesù Children’s Hospital in Rome (OPBG). The inclusion criteria for selection of controls were: (i) absence of GI infections, and (ii) no antibiotic and pre-probiotic administration in the previous two months.

Good clinical practices were followed throughout the study, in accordance with the ICH-GCP guidelines and Helsinki Declaration. Approvals were obtained from the institutional ethics committee (Ethical Committee n. 4421, “Sapienza University”) for the study protocols. Moreover, informed consent was taken from patients and controls, with the guidance of the hospital ethics committee and approval of regulatory agencies. Sample collection for GM ecological and metabolomics profiling, biobanking and integration processing of omics data was conducted at the Human Microbiome Unit OPBG and NMR-based Metabolomics Laboratory, Sapienza University of Rome.

### 3.2. Gut Microbiome-Targeted Metagenomics and Metabolomics Profiling

#### 3.2.1. Targeted Metagenomic Profiling

The general workflow included isolation of genomic DNA from stool samples, followed by amplification and sequencing of the variable region from the 16S rRNA gene, as described by Botticelli et al. [17]. DNA extraction was carried out using the QIAmp Fast DNA Stool mini kit from Qiagen, Hilden, Germany. The variable region V3-V4 from the 16S rRNA gene (~460 bp) was amplified according to the MiSeq rRNA Amplicon Sequencing workflow (Illumina, San Diego, CA, USA). For the amplicons purification, AMPure XP beads (Beckman Coulter Inc., Beverly, MA, USA) were used. A second round of amplification was conducted with a unique set of Illumina Nextera adaptor primers followed by purification to obtain the final library. The quantification was performed using Quant-iT™ PicoGreen^®^ dsDNA Assay Kit (Termo Fisher Scientifc, Waltham, MA, USA). The library was finally diluted to 4 nM, pooled and sequenced using the Illumina MiSeqDX. The Qiime v1.8. (http://qiime.org/1.4.0/) pipeline was followed [41], sequences were organized into operational taxonomic units (OTUs) and alignments were conducted using PyNAST v.0.1 software (https://biocore.github.io/pynast/) [42] against the Greengenes 13_08 database [43] with a 97% threshold of similarity.

#### 3.2.2. Metabolomic Profiling

The stool samples were further investigated to detect volatile organic compounds (VOCs) and non-VOCs [17,44].

Gas chromatography solid-phase microextraction (GC–MS/SPME) was used to characterize and quantify the VOCs into different chemical classes—alcohols, esters, aldehydes, ketones, alkenes, alkanes, etc. [44]. Samples were exposed to the SPME fiber for 45 min and then, for desorption, were placed for 10 min into the GC injection port. The GC–MS analyses were obtained using the Agilent Technologies 7890B GC coupled to a 5977A mass selective detector. The instrument was equipped with a Restek capillary column with dimensions of 60 m and 0.32 mm. Run conditions were based on the same conditions reported by Botticelli et al. [17]. The chromatograms were utilized by integration and identification of the fragment pattern with the ones present in the mass spectral library (NIST library) (version 2.2, NIST 14MS database; National Institute of Standards and Technology, Rockville, MD) and bibliography [45] and then followed by visual inspection. The data were detected by interpolating the relative area vs. internal standard (IS) area and these data were expressed as ppm (mg/kg).

Similarly, the non-VOCs in stools, i.e., acids, amino acids, amines and sugars, were analyzed, only for NSCLC patients, by NMR spectroscopy [46]. For this experiment, 500 mg of stool was suspended with 1 mL of D_2_O–PBS–NaN_3_ buffered solution. In order to obtain fecal waters, the following steps were performed. Firstly, the sample underwent vortex for 2 min and then it was centrifuged for 25 min at 10,000 rpm at 4 °C. In accordance with the study of Brasili et al., the supernatant was filtered and an entire 600 µL of volume was analyzed [46]. The NMR analysis was executed at 298 K by means of a Bruker Advance400 spectroscope (Bruker BioSpin GmbH, Rheinstetten, Germany), facilitated with a magnet operating at 9.4 Tesla (400.13 MHz for 1 H frequency). 1D ^1^H NMR experiments were carried out by implementing the standard presaturation pulse sequence. The spectral conditions and 2D homonuclear NMR total correlated spectroscopy (TOCSY) and heteronuclear single quantum coherence (HSQC) experiments were performed according to Brasili et al. [47] and Wishart et al. [48]. ACD Lab 1D-NMR Manager 12.0 software (Advanced Chemistry Development, Inc., Toronto, ON, Canada) helped to process and measure 1D NMR spectra, while MestReC software (Mestrelab Research SL, Santiago de Compostela, Spain) was used to evaluate 2D NMR spectra. The metabolites quantification was detected by the confrontation of the integrals (normalized for number of protons) of specific signals with the IS trimethylsilyl propionic acid sodium salt (TSP) and then normalized for feces weight (µmol/g).

### 3.3. Data Pre-Processing

The targeted metagenomic and metabolomic profiling was performed for two distinct groups, NSCLC and CTRLs, consisting of 11 and 8 subjects, respectively. In order to use a homogeneous approach, a unique dataset was created that included both NSCLC and CTRLs. Thereby, for each element of the two groups “*s*” (OTUs and metabolites), patients and controls were concatenated into a single array, that is, [P_i_, C_j_] (i = 1, …, 11; j = 1, …, 8).

Consequently, the data were transformed into Z scores across subjects. This means that, for each element of the two groups “*s*” and each subject “*k*”, the Z score *Z_s_*(*k*) was calculated as Equation (1)

(1) $$Z_{s}\left(k\right)=\frac{s_{k}-\sum_{k=1}^{N}s_{k}}{\frac{1}{N}\sqrt{\sum_{k=1}^{N}{\left(s_{k}-\frac{1}{N}\sum_{k=1}^{N}s_{k}\right)}^{2}}}$$

The total number of samples was denoted by *N*, where N = samples in group NSCLC + samples in group CTRLs (i.e., 11 + 8 = 19). Suitable thresholds were set with the view to remove features whose expression was nearly zero or had no significant variation across N samples. Firstly, a threshold was set to allow poorly expressed features to be equal to zero for the maximum number of samples out of N. The second threshold was set to optimize the variation, by measuring the interquartile range (IQR) percentile allowed for each gene across N samples. For the final data, species with a presence of <25% and a minimum IQR percentile of 11 were included. Finally, two additional thresholds were used to eliminate features whose expression did not vary between the two given conditions, i.e., NSCLC and CTRLs. These criteria also removed features whose expression did not change enough or changed without statistical significance [49] among NSCLC and CTRLs. To begin with, the log fold-change (the logarithm of the ratio) between the average expression of NSCLC samples and the average expression of CTRLs samples was considered. Then, a fixed cut-off value of 3.4 was set as the threshold on the log fold-change, which allowed the removal of features falling behind in absolute value.

The last threshold regarded the smallest probability (*p*-value) which allowed the rejection of the null hypothesis (i.e., the means of the two distributions—CTRLs and NSCLC—are identical) of a non-parametric permutation test. In order to adjust the statistical tests for multiple comparisons, the FDR method [50] was used for *p*-values estimation. In order to retain only significant correlations, the *p*-values associated with the correlation coefficients were corrected for multiple comparisons and a threshold was set. Accordingly, only correlations associated with *p*-values ≤ 0.05 and FDR-corrected were considered.

### 3.4. Network Analysis

The data pre-processing resulted in a threshold-set correlation matrix. The features included in the correlation matrix calculation were treated as nodes of the graph (Supplementary Tables S1 and S2) showing the identification numbers for the different features. The non-zero entries in the matrix were considered as weighted connections or edges between the pairs of nodes. Subsequently, an adjacency matrix (a representative 2D matrix whose elements indicate whether pairs of nodes are connected or not in the network) was obtained by binarizing the thresholded correlation matrix [51]. Network analysis can give us an insight into the probable role of OTUs and metabolites in terms of centrality and help deduce the importance of their participation in biological interactions. In order to illustrate the relevance of the gut metagenome and metabolome profile in NSCLC patients undergoing Nivolumab (anti-PD1) treatment, network analysis was conducted for the selected elements obtained after data pre-processing and returned only those significantly expressed in NSCLC and CTRLs, as described above.

#### 3.4.1. Degree, Betweenness Centrality and Clustering Coefficient

Centrality is a term used to estimate how important a node or edge is for the connectivity or flow of information in the network. Degree is a simple measure of centrality, corresponding to the number of edges that connect to a node (synonymous with the collector of information within the network).

Therefore, a node with a high degree will have a significant role in the network.

If A = {*a_ij_*} is the adjacency matrix, the degree centrality is given by Equation (2)

(2) $$d\left(i\right)=\sum_{k=1}^{N}a_{ki}$$

Betweenness centrality represents the capacity to connect two or more non-adjacent nodes (synonymous with influence and control over the network) and measures the extent to which a node lies on sequences of edges (paths) between other nodes [52]. Nodes with high betweenness centrality are of great interest as they control the flow of information in the network. Conversely, removal of these nodes from the network might disrupt the interaction among other nodes lying between them on the largest number of paths. The betweenness centrality is measured by how often a node occurs on all the geodesic paths (shortest paths) between two nodes. It is to be noted that a geodesic path is not necessarily unique and the geodesic paths between a single pair of nodes may pass through some of the same nodes. Mathematically, let *d_ts_*(*i*) be the number of geodesic paths from nodes “*s*” to “*t*”, that pass through “*i*”, and let *d_ts_* be the total number of geodesic paths from *s* to *t*. Then, the betweenness centrality of node “*i*” is Equation (3)

(3) $$b\left(i\right)=\sum_{\begin{array}{l} t,s=1\\ t\neq s\neq i \end{array}}^{N}\frac{d_{ts}\left(i\right)}{d_{ts}}$$

Another statistical description used in network analysis is the clustering coefficient, which represents the ability to form cores or cliques (synonymous with local communication efficiency within the network) and gives the measure of the degree to which nodes in a graph tend to cluster together [53]. The Clustering coefficient, C(i), of a node is a measure of the number of triangles connected to that node, normalized to the number of triples connected to that node [54]. A triple connected to a node is a set of two edges connected to it. If the degree of the node *i* is zero or one, we set C(i) = 0.

#### 3.4.2. Communities and Modularity

One of the major objectives of network analysis is to detect communities. Communities are a subset of nodes within a network that have dense internal connections, compared to the connections with the rest of the network [55]. If a network consists of two isolated components, each community is limited to only one component. Moreover, nodes in a community are likely to have more connections to nodes of the same community than to nodes in other communities [55]. The most common approach to community detection is based on the maximization of some modularity measure. Modularity can be defined as the quantification of the strength of the division of a network into different parts. Here, in order to find the modular properties of the network of interest, the Louvain method was used for community detection [56]. It maximizes a modularity score for each community, where the modularity quantifies the quality of the assignment of nodes to a particular community by evaluating how many connections the nodes have within a community, compared to the number of connections they would have in a random network.

### 3.5. Weighted Gene Co-Expression Network Analysis (WGCNA)

WGCNA is the most commonly employed method to construct gene co-expression networks, identifying modules and detecting the central or hub genes within the modules [22,23]. The WGCNA R package consists of the functions used to perform the co-expression network analysis [23]. Here, the WGCNA framework was applied on the merged dataset (metagenomics and metabolomics) to identify features (OTUs and metabolites) with similar profiles in the sample population.

The objective was to identify modules that are associated with two traits of interest: (i) condition trait, i.e., NSCLC or CTRLs status, and (ii) treatment trait, i.e., R or NR. Sample clustering was conducted to detect outliers and to visualize how the traits (i.e., NSCLC/CTRLs status and R/NR status) would relate to the sample dendrogram.

Subsequently, a weighted network was built by computing the Pearson correlation coefficient between any pair of nodes and by setting a soft-thresholding power equal to 5. The choice of this correlation threshold was based on two criteria.

The first selection criterion derived from the evidence that most biological networks show a scale-free distribution of the node degree [57,58,59]. Hence, the obtained network should have approximately this topology. Moreover, scale-free networks are highly heterogeneous and their topology is dominated by hubs, which are the few highly connected nodes. These hubs link the rest of the less connected nodes within the network. One of the defining properties of scale-free networks is that the degree distribution P(k) (i.e., the probability that a node has k edges) decays as a power law P(k)~k^(−α)^. Therefore, in order to evaluate whether the correlation network displays a scale-free behavior, the index R-squared (defined as the square of the correlation between log(P(k)) and log(k)) was calculated as a function of the correlation threshold. As it is not biologically reasonable that a network includes more hubs than non-hubs, R-squared was multiplied with −1. If the estimated coefficient α of the best fitting regression line between log(P(k)) and log(k) is positive, then a signed version of this index is obtained. However, if the R-squared approaches 1, then there is a linear relationship between log(P(k)) and log(k) and a scale-free topology is attained. Overall, these points were taken into consideration so as to select a correlation threshold able to guarantee, at least approximately, a scale-free network topology, i.e., signed R-squared >0.8 [22].

The second criterion allowed selecting a threshold that would guarantee a reasonable mean connectivity.

At the end, WGCNA produced a set of modules (labeled by color), each containing a set of unique nodes (OTUs and metabolites).

To summarize the information contained in a given module of the WGCNA network, the module eigengene (ME) was used. ME is defined as the first principal component of the module [23]. Accordingly, the ME can be defined as a representative node capable of condensing each module into a single profile. Next, the module membership (MM) and the biomarker significance (BS) were calculated for each node in each module [23]. The MM of a node represents the correlation between the node profile and the ME, whereas the BS of a node represents the correlation between the node profile and a given trait. If the MM of a node is close to 0, the node is not considered a part of the module. Conversely, if the MM tends to 1 or −1, the node is highly representative of the module. The sign of the MM explains if the node has a positive or a negative relationship with the ME. Concerning the BS, the greater the absolute value of the BS of a node, the more biologically significant the node is. A BS of 0 indicates that the node is not significant with reference to the trait of interest.

## 4. Conclusions

In 2013, science named immunotherapy as a breakthrough in cancer treatment that demonstrated promising results bettering the prognosis of cancer patients across the globe [60,61]. The success was attained through ICIs and chimeric antigen receptor (CAR) T cells [61]. As described by Chen and Mellman, the cancer immunity cycle is now known to play an important role in the identification and utilization of checkpoint inhibitor targets [4]. Recently, several studies suggested the role of the GM in modulating patient response to immunotherapy in cancer [16,17,36,62,63]. This evidence also implied that certain host bacteria in the gut contributed to the positive response rate to immunotherapies, while others exhibited the opposite effect. Such findings emphasized that each individual’s microbiome is unique and hence immunotherapy response may vary due to the composition of the GM at the time of immunotherapy.

Here, we have focused on the impact of the GM on NSCLC and the efficacy of ICIs in the treatment. Preliminary data by Botticelli and colleagues suggested an important role of GM metabolic pathways in immunotherapy response [17]. Their findings linked the presence of SCFAs as an important indicator of positive response to treatment. Such discoveries of microbiota-linked indicators of non-responder and responder patients can be a positive indication on the road to personalized management of cancer patients.

Here, a network approach was used in order to gain a deeper understanding of the crosstalk between the GM and lungs. Using network degree and centrality measures, it was found that metabolites like indole 3-methyl, butanal 3-methyl, indole, pentanol and heptane could be some of the main signaling molecules regulating gut ecosystem functioning and, thus, affecting host health. WGCNA generated strong correlations between the presence of SCFAs and a normal healthy GM, supporting Botticelli et al.’s hypothesis [17], and therefore it is potentially possible to consider SCFAs as biomarkers of anti-PD1 therapy response in NSCLC patients. Furthermore, commensals such as *Akkermansia muciniphila*, Rikenellaceae, *Bacteroides*, Peptostreptococcaceae, Mogibacteriaceae and Clostridiaceae were found to be more abundant in controls but not in lung cancer patients, thus proposing an imbalance in the intestinal microbiota of NSCLC patients. Interestingly, *Granulicatella* showed a significant correlation with anti-PD1 treatment responders, and hence could be a choice for diagnostic and prognostic biomarkers. Hence, not only the differences in microbial ecology could explain the different responses to therapy but it is also necessary to take into account the microbial functional activity of the entire community, to understand which signaling molecules are employed for a crosstalk with host. In this experimental study, molecules such as indoles and aldehydes emerged as particularly noteworthy for the signaling role in GM functionality.

Moving forward, it is necessary to conduct further investigations on larger datasets to unravel the complex relationship between gut microorganisms, metabolites, tumor pathogenesis and host immunity. The gut–lung microbiota axis could influence ICIs treatment response in NSCLC and other cancers. The mechanisms by which the microorganisms in the gut and various other body sites, individually or collectively, interact with the host immune system is still largely unknown.

Studies also highlighted fecal microbiota transplantation (FMT) as a new promising therapeutic strategy to treat cancer, which involves complex microbiota community transfers, dietary prebiotics and elimination of harmful gut microbes with selective antibiotics or viruses [64,65,66].

To conclude, further research in this direction is necessary to improve patients’ treatment responses and life conditions, through new therapeutic approaches to cancer.

## Supplementary Materials

Supplementary Materials can be found at https://www.mdpi.com/1422-0067/21/22/8730/s1. Supplementary Text S1. Results of Weighted Gene Co-expression Network Analysis (WGCNA)_GC_NMR_OTU; Supplementary Figure S1. Weighted Gene Co-expression Network Analysis (WGCNA) analysis on GC-NMR-OTU data; Supplementary Figure S2. Visualization of Weighted Gene Co-expression Network Analysis (WGCNA) network on GC-NMR-OTU data; Supplementary Figure S3. Abundance of the 44 selected features; Supplementary Table S1. OTUs raw data; Supplementary Table S2. GC–MS raw data; Supplementary Table S3. NMR raw data; Supplementary Table S4. Weighted Gene Co-expression Network Analysis (WGCNA) modules OTUs NMR GC–MS; Supplementary Table S5. Weighted Gene Co-expression Network Analysis (WGCNA) analysis; Supplementary Table S6. Statistics of the 44 selected features; Supplementary Table S7. List of identification numbers for the OTUs + metabolites network.

## Abbreviations

GM	Gut microbiome
GI	Gastrointestinal
CTL-4	Cytotoxic T-Lymphocyte Antigen 4
PD-1	Programmed cell death protein 1
PD-L1	Programmed death-ligand 1
ICIs	Immune checkpoint inhibitors
PRR	Pattern recognition receptors
NSCLC	Non-small cell lung cancer
SCFAs	Short chain fatty acids
WGCNA	Weighted Gene Co-expression Network Analysis
ECOG	Eastern Cooperative Oncology Group
i-RECIST	Response Evaluation Criteria in Solid Tumors
PFS	Progression-free survival
OS	Overall survival
NR	Non-responders
R	Responders
CTRLs	Controls
OTUs	Operational taxonomic units
VOCs	Volatile organic compounds
GC-MS/	Gas-chromatography-mass spectrometry/
SPME	Solid phase microextraction/
IS	Internal Standard
NMR	Nuclear magnetic resonance spectroscopy
TSP	Trimethylsilyl propionic acid sodium salt
IQR	Inter Quartile Range
FDR	False Discovery Rate
ME	Module eigengene
MM	Module membership
BS	Biomarker significance
CAR	Chimeric antigen receptor
FMT	Fecal microbiota transplantation
