# Supplementary material for: Network Analysis of Gut Microbiome and Metabolome to Discover Microbiota-Linked Biomarkers in Patients Affected by Non-Small Cell Lung Cancer
Source: Int J Mol Sci. 2020 Nov 19;21(22):8730. doi: 10.3390/ijms21228730 (PMC7699235; doi:10.3390/ijms21228730)
Supplement: Supplementary file 1 [file ijms-21-08730-s001.zip › Supplementary R1/Supplementary Figure S3_R1.docx]

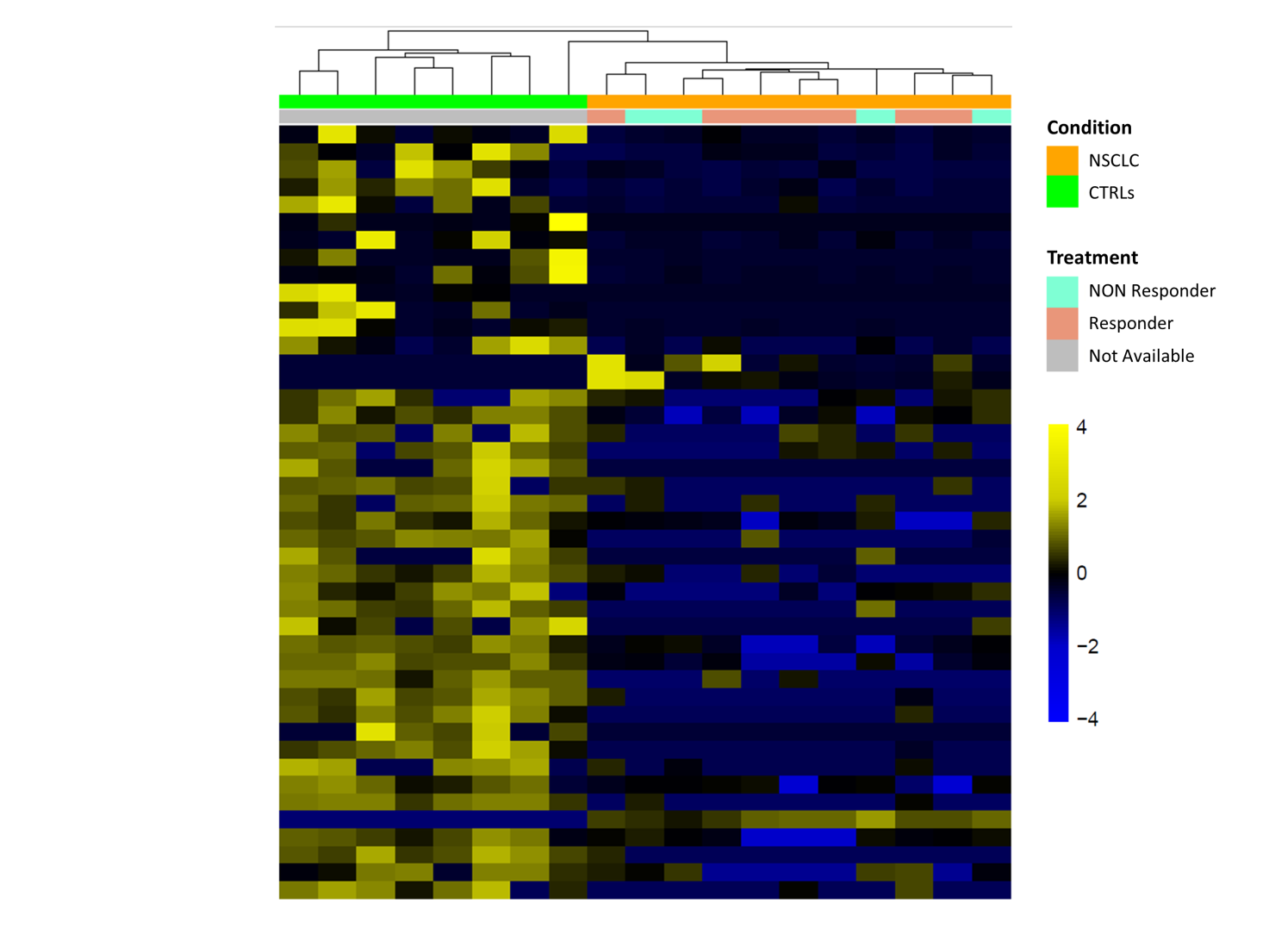


**Figure S3. Abundance of the 44 selected features.** Heatmap representing the 44 features (OTUs and metabolites) selected at the end of the data pre-processing (rows) across all samples (columns). Data are log2-transformed and z-score normalized. The 44 features are clustered according to samples along the columns by using the Euclidean distance as metrics. The sample labels are coloured according to clinical status (Condition) and response to therapy (Treatment). Heatmap colors represent different expression levels increasing from blue to yellow.
