# Supplementary material for: Network Analysis of Gut Microbiome and Metabolome to Discover Microbiota-Linked Biomarkers in Patients Affected by Non-Small Cell Lung Cancer
Source: Int J Mol Sci. 2020 Nov 19;21(22):8730. doi: 10.3390/ijms21228730 (PMC7699235; doi:10.3390/ijms21228730)
Supplement: Supplementary file 1 [file ijms-21-08730-s001.zip › Supplementary R1/Supplementary Table S6_R1.docx]

| **Features** | **logFC**  **(NSCLC/ CTRL)** | **p-value** | **FDR** |
| --- | --- | --- | --- |
| k__Bacteria;p__Firmicutes;c__Clostridia;o__Clostridiales;f__Peptostreptococcaceae;g__ | -0.022 | 5.29E-05 | 0.001045 |
| k__Bacteria;p__Proteobacteria;c__Betaproteobacteria;o__Burkholderiales;f__Comamonadaceae;g__Comamonas | 0.024078 | 0.00019 | 0.001045 |
| Butyric acid | -11.3197 | 0.000146 | 0.001045 |
| Caryophyllene | -7.89366 | 0.000148 | 0.001045 |
| g-Terpinene | -6.63898 | 0.000103 | 0.001045 |
| Indole, 3-methyl- | -10.3712 | 0.000146 | 0.001045 |
| Methyl Isobutyl Ketone | 6.000548 | 0.00019 | 0.001045 |
| Pentanoic acid | -10.974 | 0.000103 | 0.001045 |
| 3-Carene | -6.337 | 0.000292 | 0.001175 |
| Benzaldehyde | -7.24252 | 0.00032 | 0.001175 |
| Benzeneacetaldehyde | -5.94487 | 0.000309 | 0.001175 |
| Butanal, 3-methyl- | -7.53733 | 0.000292 | 0.001175 |
| 1-Pentanol | -4.03266 | 0.000439 | 0.001387 |
| 6-Methyl-5-hepten-2-one | -8.90112 | 0.000441 | 0.001387 |
| Acetone | -7.10121 | 0.000587 | 0.001722 |
| k__Bacteria;p__Firmicutes;c__Clostridia;o__Clostridiales;f__Peptococcaceae;g__Desulfitobacter | 0.006533 | 0.00082 | 0.002255 |
| k__Bacteria;p__Firmicutes;c__Clostridia;o__Clostridiales;f__[Mogibacteriaceae];g__ | -0.00439 | 0.001191 | 0.002758 |
| 2-Butenal | -6.00792 | 0.001143 | 0.002758 |
| 2-Octanone | -4.05314 | 0.001086 | 0.002758 |
| 2-Hexanone | -4.89149 | 0.001441 | 0.003171 |
| Pyridine | -4.08251 | 0.001778 | 0.003725 |
| 2-Butanone | -5.20399 | 0.002011 | 0.004022 |
| 2-Heptanone | -4.3746 | 0.002598 | 0.004971 |
| Indole | -4.09865 | 0.003361 | 0.005916 |
| p-Cresol | -6.24622 | 0.00332 | 0.005916 |
| Anethole | -5.04281 | 0.003617 | 0.006121 |
| Butanoic acid, ethyl ester | -4.21985 | 0.004026 | 0.006561 |
| k__Bacteria;p__Bacteroidetes;c__Bacteroidia;o__Bacteroidales;f__Bacteroidaceae;g__Bacteroidesuniformis | -0.02242 | 0.004975 | 0.007548 |
| k__Bacteria;p__Firmicutes;c__Clostridia;o__Clostridiales;f__Lachnospiraceae;g__Roseburiafaecis | -0.00234 | 0.004975 | 0.007548 |
| Acetoin | -5.52621 | 0.007278 | 0.010675 |
| k__Archaea;p__Euryarchaeota;c__Methanobacteria;o__Methanobacteriales;f__Methanobacteriaceae;g__Methanobrevibacter | -0.06589 | 0.008206 | 0.011647 |
| k__Bacteria;p__Actinobacteria;c__Coriobacteriia;o__Coriobacteriales;f__Coriobacteriaceae;g__ | -0.01459 | 0.009103 | 0.012137 |
| k__Bacteria;p__Actinobacteria;c__Coriobacteriia;o__Coriobacteriales;f__Coriobacteriaceae;g__Collinsellaaerofaciens | -0.01697 | 0.009103 | 0.012137 |
| k__Bacteria;p__Actinobacteria;c__Actinobacteria;o__Bifidobacteriales;f__Bifidobacteriaceae;g__Bifidobacteriumpseudolongum | -7.52E-05 | 0.010059 | 0.013017 |
| k__Bacteria;p__Bacteroidetes;c__Bacteroidia;o__Bacteroidales;f__Bacteroidaceae;g__Bacteroidescaccae | -0.00206 | 0.012066 | 0.014748 |
| k__Bacteria;p__Bacteroidetes;c__Bacteroidia;o__Bacteroidales;f__Rikenellaceae;g__ | -0.02036 | 0.012066 | 0.014748 |
| 2,3-Butanedione | -3.85859 | 0.01285 | 0.015281 |
| 6-Methyl-3,5-heptadiene-2-one | -4.41857 | 0.018676 | 0.021625 |
| 1-Hexanol | -3.15695 | 0.030827 | 0.034779 |
| k__Bacteria;p__Actinobacteria;c__Coriobacteriia;o__Coriobacteriales;f__Coriobacteriaceae;g__Atopobium | -0.00062 | 0.032462 | 0.035708 |
| Heptane, 3,4-dimethyl- | -5.18462 | 0.038629 | 0.041455 |
| Phenol | -4.02253 | 0.04125 | 0.043215 |
| k__Bacteria;p__Firmicutes;c__Clostridia;o__Clostridiales;f__Ruminococcaceae;g__Ruminococcusbromii | -0.0134 | 0.057434 | 0.058769 |
| k__Bacteria;p__Verrucomicrobia;c__Verrucomicrobiae;o__Verrucomicrobiales;f__Verrucomicrobiaceae;g__Akkermansiamuciniphila | -0.09941 | 0.062025 | 0.062025 |

**Table S6. Statistics of the 44 selected features.** The table reports the list of the 44 selected features (OTUs and metabolites) with their associated logarithmic fold-changes (logFC) and p-values. P-values were computed from the Wilcoxon rank sum test and adjusted according to the False Discovery Rate (FDR) method.
