# Supplementary material for: Network Analysis of Gut Microbiome and Metabolome to Discover Microbiota-Linked Biomarkers in Patients Affected by Non-Small Cell Lung Cancer
Source: Int J Mol Sci. 2020 Nov 19;21(22):8730. doi: 10.3390/ijms21228730 (PMC7699235; doi:10.3390/ijms21228730)
Supplement: Supplementary file 1 [file ijms-21-08730-s001.zip › Supplementary R1/Supplementary Figure S1_NMR_GC_OTUs.doc]

**Figure S1. Weighted Gene Co-expression Network Analysis (WGCNA) analysis on GC-NMR-OTU data.** a) *Clustering dendrogram of samples*. Sample clustering was conducted to detect outliers. All samples are located in the clusters and passed the cutoff thresholds. The horizontal bars represent how the treatment trait relate to the sample dendrogram: white (low value) represents not-responder (NR), whereas red (high value) represents responder (R). b) *Calculation and selection of optimal soft-thresholding power*. Influence of different powers on the scale independence (left) and on the mean connectivity (right). The selected soft-thresholding power is indicated by a red arrow. c) *Barplot*. The bars represent the size of each WGCNA detected module and are colored according to the corresponding module labels. d) *Module-trait associations*. In the heatmap, each row corresponds to a module eigengene and each cell contains the corresponding correlation and p-value with the condition R/NR. The table is color-coded by correlation according to the color legend.
