# Supplementary material for: Network Analysis of Gut Microbiome and Metabolome to Discover Microbiota-Linked Biomarkers in Patients Affected by Non-Small Cell Lung Cancer
Source: Int J Mol Sci. 2020 Nov 19;21(22):8730. doi: 10.3390/ijms21228730 (PMC7699235; doi:10.3390/ijms21228730)
Supplement: Supplementary file 1 [file ijms-21-08730-s001.zip › Supplementary R1/Supplementary Figure S2_ NMR_GC_OTUs.doc]

**Figure S2**. **Visualization of Weighted Gene Co-expression Network Analysis (WGCNA) network on GC-NMR-OTU data**. Weighted Correlation Network [UPPER] and characterization of turquoise module [BOTTOM]. In the network, the WGCNA detected module are colored according to the corresponding module labels. Pie charts represent the percentages and numbers of OTUs and metabolites falling in turquoise module. In the subnetwork of turquoise module the species with the highest BS are highlighted with a greater node size and colored in red, if increase in R samples, or in blue, if increase in NR samples.
