# Supplementary material for: Network Analysis of Gut Microbiome and Metabolome to Discover Microbiota-Linked Biomarkers in Patients Affected by Non-Small Cell Lung Cancer
Source: Int J Mol Sci. 2020 Nov 19;21(22):8730. doi: 10.3390/ijms21228730 (PMC7699235; doi:10.3390/ijms21228730)
Supplement: Supplementary file 1 [file ijms-21-08730-s001.zip › Supplementary R1/Supplementary Table S7_features_R1.doc]

| **Supplementary Table S7**. List of Identification Numbers for the OTUs and Metabolites Network | |
| --- | --- |
| **Sl#** | **Species** |
| 1 | 'p__Actinobacteria;c__Coriobacteriia;o__Coriobacteriales;f__Coriobacteriaceae;g__' |
| 2 | 'p__Bacteroidetes;c__Bacteroidia;o__Bacteroidales;f__Bacteroidaceae;g__Bacteroidescaccae' |
| 3 | 'p__Bacteroidetes;c__Bacteroidia;o__Bacteroidales;f__Bacteroidaceae;g__Bacteroidesuniformis' |
| 4 | 'p__Bacteroidetes;c__Bacteroidia;o__Bacteroidales;f__Rikenellaceae;g__' |
| 5 | 'p__Verrucomicrobia;c__Verrucomicrobiae;o__Verrucomicrobiales;f__Verrucomicrobiaceae;g__Akkermansiamuciniphila' |
| 6 | 'p__Actinobacteria;c__Coriobacteriia;o__Coriobacteriales;f__Coriobacteriaceae;g__Collinsellaaerofaciens' |
| 7 | 'k__Bacteria;p__Firmicutes;c__Clostridia;o__Clostridiales;f__Lachnospiraceae;g__Roseburiafaecis' |
| 8 | 'k__Bacteria;p__Firmicutes;c__Clostridia;o__Clostridiales;f__Peptostreptococcaceae;g__' |
| 9 | 'k__Bacteria;p__Firmicutes;c__Clostridia;o__Clostridiales;f__[Mogibacteriaceae];g__' |
| 10 | 'p__Euryarchaeota;c__Methanobacteria;o__Methanobacteriales;f__Methanobacteriaceae;g__Methanobrevibacter' |
| 11 | 'p__Firmicutes;c__Clostridia;o__Clostridiales;f__Ruminococcaceae;g__Ruminococcusbromii' |
| 12 | 'p__Actinobacteria;c__Coriobacteriia;o__Coriobacteriales;f__Coriobacteriaceae;g__Atopobium' |
| 13 | 'p__Actinobacteria;c__Actinobacteria;o__Bifidobacteriales;f__Bifidobacteriaceae;g__Bifidobacteriumpseudolongum' |
| 14 | 'p__Firmicutes;c__Clostridia;o__Clostridiales;f__Peptococcaceae;g__Desulfitobacter' |
| 15 | 'p__Proteobacteria;c__Betaproteobacteria;o__Burkholderiales;f__Comamonadaceae;g__Comamonas' |
| 16 | '1-Hexanol' |
| 17 | '1-Pentanol' |
| 18 | '2.3-Butanedione' |
| 19 | '2-Butanone' |
| 20 | '2-Butenal' |
| 21 | '2-Heptanone' |
| 22 | '2-Hexanone' |
| 23 | '2-Octanone' |
| 24 | '3-Carene' |
| 25 | '6-Methyl-3.5-heptadiene-2-one' |
| 26 | '6-Methyl-5-hepten-2-one' |
| 27 | 'Acetoin' |
| 28 | 'Acetone' |
| 29 | 'Anethole' |
| 30 | 'Benzaldehyde' |
| 31 | 'Benzeneacetaldehyde' |
| 32 | 'Butanal. 3-methyl-' |
| 33 | 'Butyric acid' |
| 34 | 'Caryophyllene' |
| 35 | 'Butanoic acid. ethyl ester' |
| 36 | 'g-Terpinene' |
| 37 | 'Heptane. 3.4-dimethyl-' |
| 38 | 'Indole' |
| 39 | 'Indole. 3-methyl-' |
| 40 | 'Methyl Isobutyl Ketone' |
| 41 | 'p-Cresol' |
| 42 | 'Pentanoic acid' |
| 43 | 'Phenol' |
| 44 | 'Pyridine' |
