# Supplementary material for: Network Analysis of Gut Microbiome and Metabolome to Discover Microbiota-Linked Biomarkers in Patients Affected by Non-Small Cell Lung Cancer
Source: Int J Mol Sci. 2020 Nov 19;21(22):8730. doi: 10.3390/ijms21228730 (PMC7699235; doi:10.3390/ijms21228730)
Supplement: Supplementary file 1 [file ijms-21-08730-s001.zip › Supplementary R1/Supplemtary Text S1_Results NMR GC OTus.doc]

**Supplementary Text S1. Results of Weighted Gene Co-expression Network Analysis (WGCNA) network ON OTU-GC-NMR**

Sample clustering was conducted to detect outliers and to visualize how the treatment trait relate to the sample dendrogram (**Figure S1a**). A weighted network (soft-thresholding) was built by using the Pearson correlation metrics, a minimum module size of 30, and a soft-thresholding power equal to 9. This choice for the correlation items from two selection criteria, i.e. to choose a correlation threshold that i) can lead to a network satisfying scale-free topology at least approximately and that ii) guarantees a reasonable mean connectivity (**Figure S1b**).

The final network consisted of 6 modules (labeled by color), ranging in size from 31 to 70 members (**Figure S1c**). The grey module is a grouping of nodes with outlying profiles and was not considered further.

To summarize all the information contained in a given module of the Weighted Gene Co-expression Network Analysis (WGCNA) network, we used the module eigengene (ME) defined as the first principal component of the module [1]. Thus, the ME can be considered a representative node able to condense each module into one profile. Then, for each node in a given module, the module membership (MM) and the biomarker significance (BS) can be computed [1]. The MM of node represents the correlation between the node profile and the ME, whereas the BS of node represents the correlation between the node profile and a given trait. If the MM of a node is close to 0, the node is not part of the module. On the other hand, if the MM is close to 1 or -1, the node is highly connected to the module. The sign of the MM encodes whether the node has a positive or a negative relationship with the ME. On the other hand, the higher the absolute value of the BS of a node, the more biologically significant is the node. A gene significance of 0 indicates that the node is not significant with regard to the trait of interest. For each node in each module, the values of MM and BS and the corresponding p-values are reported in **Supplementary Table S4.**

Eventually, test of association between treatment trait and the module eigengenes was performed for each module and the results were summarized in a heatmap (**Figure S1d**).

We found only one module, i.e. the turquoise module, with a statistically significant (p-value <0.1) correlation with respect to the NR/R trait (**Figure S1d**). The positive sign of the correlation value means that the “species” (OTUs and metabolites) falling in this module are mainly expressed in R with respect to NR.

In detail, the turquoise module includes a total of 70 species, including 30 OTUs and 40 metabolites (**Figure S2**).By ranking these species according to their BS, we found that the most interesting species with respect to the treatment trait are Taurine, Propionate, Lysine, Glutamate and Lactacte+Threonine (highest positive BS and thus up-regulated in responder) and Dodecane, Benzeneacetaldehyde, 2-Octanone, 2-Pentanone and Tridecane (lowest negative BS) and thus up-regulated in NR.

**References**

1.	Langfelder, P.; Horvath, S. WGCNA: an R package for weighted correlation network analysis*. BMC Bioinformati*c**s 20**08*,* 9, 559, doi:10.1186/1471-2105-9-559.
